# Optimal number of stimulation contacts for coordinated reset neuromodulation

**DOI:** 10.3389/fneng.2013.00005

**Published:** 2013-07-22

**Authors:** Borys Lysyansky, Oleksandr V. Popovych, Peter A. Tass

**Affiliations:** ^1^Institute of Neuroscience and Medicine – Neuromodulation (INM-7), Research Center JuelichJuelich, Germany; ^2^Department of Neuromodulation, University of CologneCologne, Germany

**Keywords:** coordinated reset stimulation, neuronal synchronization, electrical stimulation, parameter optimization, synchronization control

## Abstract

In this computational study we investigate coordinated reset (CR) neuromodulation designed for an effective control of synchronization by multi-site stimulation of neuronal target populations. This method was suggested to effectively counteract pathological neuronal synchrony characteristic for several neurological disorders. We study how many stimulation sites are required for optimal CR-induced desynchronization. We found that a moderate increase of the number of stimulation sites may significantly prolong the post-stimulation desynchronized transient after the stimulation is completely switched off. This can, in turn, reduce the amount of the administered stimulation current for the intermittent ON–OFF CR stimulation protocol, where time intervals with stimulation ON are recurrently followed by time intervals with stimulation OFF. In addition, we found that the optimal number of stimulation sites essentially depends on how strongly the administered current decays within the neuronal tissue with increasing distance from the stimulation site. In particular, for a broad spatial stimulation profile, i.e., for a weak spatial decay rate of the stimulation current, CR stimulation can optimally be delivered via a small number of stimulation sites. Our findings may contribute to an optimization of therapeutic applications of CR neuromodulation.

## 1. Introduction

Synchronization plays a fundamental role in many interacting systems (Winfree, 1980; Kuramoto, 1984; Tass, 1999; Pikovsky et al., 2001; Strogatz, 2003). However, pathological synchronization is a hallmark of some neurological disorders, e.g., Parkinson's disease (PD) or essential tremor (Lenz et al., 1994; Levy et al., 2002; Timmermann et al., 2003; Hammond et al., 2007; Amtage et al., 2008; Smirnov et al., 2008). Nowadays, high-frequency (HF, >100 Hz) electrical deep brain stimulation (DBS) is widely applied for the treatment of PD (Benabid et al., 1991; Blond et al., 1992) in patients who have inadequate therapeutic response to medication or have intolerable side effects from it. Mechanism of HF DBS is not fully understood yet, it may significantly modulate the neuronal firing by, e.g., suppressing or overacting it (Beurrier et al., 2001; Hashimoto et al., 2003; Filali et al., 2004; McIntyre et al., 2004). However, HF DBS may be ineffective or lead to side effects, and the clinical effect may decline with time (Limousin et al., 1999; Kumar et al., 2003; Volkmann, 2004; Rodriguez-Oroz et al., 2005), which motivated the development of novel stimulation methods (Tass, 1999). They are aimed at the control of undesirable neuronal synchronization, which is highlighted by the finding that the physiological dynamics of neuronal populations is characterized by uncorrelated firing (Nini et al., 1995). During the last decade several desynchronizing techniques for DBS have been developed with the methods of non-linear dynamics (Tass, 1999, 2003a,b; Rosenblum and Pikovsky, 2004; Hauptmann et al., 2005; Popovych et al., 2005, 2006; Pyragas et al., 2007), which provide mild, but, nevertheless, effective means for the control of pathological neuronal synchronization (Tass et al., 2006). The main distinction of the novel methods in comparison to HF DBS is that coordinated reset (CR) stimulation (Tass, 2003a,b) as well as feedback techniques (Rosenblum and Pikovsky, 2004; Hauptmann et al., 2005; Popovych et al., 2005; Pyragas et al., 2007) selectively counteract pathological synchronization of neuronal target populations and restore uncorrelated neuronal firing.

In this paper we consider CR stimulation (Tass, 2003a,b). Its mechanism of action is based on the phase reset of neuronal oscillations where electrical stimuli or synaptic input of sufficient strength reset the phase of a neuron (Winfree, 1977; Best, 1979; Tass, 1999; Popovych and Tass, 2012). After the stimulation the neuronal oscillations restart from a preferred phase. According to the CR stimulation protocol, the population of synchronized neurons is stimulated via a few stimulation sites in a timely coordinated manner. The entire neuronal population is divided into several sub-populations by CR stimulation where the phases of the neuronal oscillations of the sub-populations get phase-shifted with respect to each other, and the total synchronization is replaced by, e.g., a cluster state (Tass, 2003a,b; Lysyansky et al., 2011). Due to the pathologically strong synaptic connectivity, the entire target population runs from the cluster state through a transient characterized by pronounced desynchronization and finally resynchronizes if left unperturbed. Accordingly, to keep the neuronal ensemble in a desynchronized state, CR stimuli are delivered intermittently (Tass, 2003a,b), for instance, by applying CR in an *m* : *n* ON–OFF mode, where *m* cycles with CR are followed by *n* cycles without any stimulation (Lysyansky et al., 2011). Such a stimulation protocol has computationally been found to be effective in inducing transient desynchronization in the stimulated neuronal ensembles (Tass, 2003a,b; Lysyansky et al., 2011).

The desynchronizing effect of CR stimulation has been analyzed in several modeling papers (Tass, 2003a,b; Tass and Majtanik, 2006; Hauptmann and Tass, 2007, 2009; Tass and Hauptmann, 2007, 2009; Popovych and Tass, 2012). The CR-induced long-lasting desynchronization has experimentally been confirmed in an *in vitro* study in rat hippocampal slices (Tass et al., 2009). In addition, CR neuromodulation was tested in the 1-methyl-4-phenyl-1,2,3,6-tetrahydropyridine (MPTP)-treated macaque monkeys, the best characterized model of experimental parkinsonism, played an important role in the development of stereotactic treatments of PD (Bergman et al., 1990; Benazzouz et al., 1993; Nini et al., 1995; Hammond et al., 2007). CR neuromodulation of the subthalamic nucleus (STN) turned out to have sustained long-lasting after-effects on motor function in MPTP monkeys (Tass et al., 2012b). In contrast, long-lasting after-effects were not observed with classical HF DBS (Tass et al., 2012b). The clinical and preclinical studies performed so far have not revealed adverse effects of CR neuromodulation (Tass et al., 2012a,b).

In clinical applications of CR neuromodulation optimal therapeutic effects should be achieved with a minimal amount of stimulation current, which can prevent from unnecessarily strong perturbation of physiological neuronal activity in the target population and spread of the stimulation current in the neuronal tissue and affecting, in such a way, neighboring regions. This can, in turn, prevent from possible side effects. In addition, high stimulation amplitudes lead to faster battery depletion of the stimulator, and prolonging battery life is a desirable outcome of parameter optimization, too. Apart from the stimulation strength, the number of stimulation contacts (for a given electrode topology, e.g., linear vs. circular alignment of stimulation contacts) is another stimulation parameter that is central to the outcome of CR neuromodulation. Accordingly, in this study we consider the impact of the number of stimulation sites on the desynchronizing effect of CR stimulation. This problem is strongly connected to the clinically important problem of the optimal design of DBS electrodes. For example, in a computational study Butson and McIntyre (2006) suggested improvements of the contact form by using finite-element models of the electrode and surrounding medium. In this way the volume of the neuronal tissue activated by DBS stimulation can effectively be controlled, which depends on many factors such as electrode impedance and capacitance, voltage drop at the electrode-tissue interface, and tissue properties (Butson et al., 2006; Chaturvedi et al., 2010).

In the present paper we show that for a linear arrangement of stimulation sites the optimal number of stimulation sites for CR stimulation essentially depends on the signal decay rate with distance from the stimulation site. On the one hand, for a rapid decay of the stimulation signal in the neuronal tissue the desynchronizing effect of CR stimulation can be improved by additional stimulation sites. On the other hand, CR stimulation of a medium with a broad spatial signal spread is optimally delivered via a small number of stimulation sites. The results are illustrated on three different oscillatory ensembles: the Kuramoto system of coupled phase oscillators, a population of FitzHugh–Nagumo (FHN) spiking neurons coupled via excitatory chemical synapses, and a network of synaptically coupled adaptive exponential integrate-and-fire bursting neurons. We also discuss the application of our findings to the clinically used Medtronic leads no. 3387 and no. 3389 DBS electrodes.

## 2. Materials and methods

### 2.1. Coupled phase oscillators

In this section we introduce a network of coupled phase oscillators as a simple model of a neuronal ensemble subjected to CR stimulation. We consider the well-known Kuramoto system of *N* all-to-all (or globally) coupled phase oscillators, where each oscillator receives the coupling from the other *N* − 1 oscillators, and which reflects several main synchronization properties of a number of oscillatory networks (Kuramoto, 1984; Acebrón et al., 2005),

(1) $${\dot{\theta }}_{j}=\omega_{j}+\frac{C}{N}\sum_{k=1}^{N}\sin(\theta_{k}-\theta_{j})+S_{j}(t),\;j=1,\;2,\;\ldots ,\;N,$$

where θ_*j*_(*t*) are the phases, ω_*j*_ are the natural frequencies, *C* is the coupling strength in the ensemble, and *S*_*j*_(*t*) is the stimulation signal which will be defined below. We consider *N* = 400 oscillators and coupling *C* = 0.1. The natural frequencies ω_*j*_ are Gaussian distributed with mean ω_mean_ = π and standard deviation σ_ω_ = 0.02. For numerical integration we use a Runge–Kutta method of order 5(4) with adaptive step size (Hairer et al., 1993).

Equation (1) governs the time-dependent dynamics of the oscillator phases θ_*j*_(*t*), *t* ≥ 0, which, in the absence of stimulation (*S*_*j*_(*t*) ≡ 0), have been found to spontaneously synchronize for sufficiently strong coupling (Kuramoto, 1984; Strogatz, 2000). In that case, a large group of oscillators starts to oscillate at the same frequency, and their phases get narrowly distributed by forming a single phase cluster, in this way constituting an in-phase synchronization. The onset of in-phase synchronization in ensemble (Equation 1) is reflected by an increasing amplitude of the mean field, which is given by the first order parameter *R*_1_ defined as $R_{m}=\left|N^{-1}\sum_{j=1}^{N}\exp(im\theta_{j})\right|$ for *m* = 1 (Haken, 1983; Kuramoto, 1984; Tass, 1999). Accordingly, large values of *R*_1_, 0 ≤ *R*_1_ ≤ 1, close to 1 correspond to in-phase synchronization of oscillators (Equation 1), whereas a desynchronized state, where the phases θ_*j*_ are uniformly distributed on the unit circle, is indicated by small values of all order parameters *R*_*m*_, *m* ≥ 1. For example, the time-averaged first order parameter 〈*R*_1_〉 ≈ 0.98 for the considered coupling strength and distribution of the natural frequencies. The above order parameters will thus be used below to characterize the extent of synchronization in ensemble (Equation 1). The order parameters *R*_*m*_ can also reflect the formation of symmetric cluster states in ensemble (Equation 1), where the phases θ_*j*_ (mod 2π) are divided into a few synchronized groups (clusters) of equal size being equidistantly spaced on a circle of length 2π. Such a cluster state comprising *l* clusters is characterized by large values of *R*_l_ combined with small values of *R*_*m*_, 1 ≤ *m* < *l* (see, for example, the values of *R*_1_ and *R*_4_ in Figure 2 below for a CR-induced 4-cluster state).

We note here that since model (Equation 1) is dimensionless, all parameters are also considered dimensionless and will, thus, be measured and illustrated in figures below in arbitrary units. We are, however, able to compare the obtained results with realistic setups and provide a mapping between the parameter space of the model and experimentally measured quantities (see section 4).

### 2.2. CR stimulation

**Figure 1:**
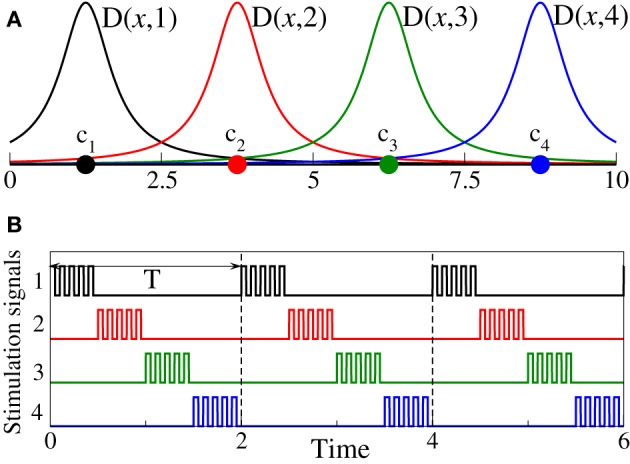
**(A)** Schematic illustration of the spatial profiles $D(x,\;k),\;k=\bar{1,\;4}$ from Equations (2, 3) of the current spread in the stimulated neuronal population for the case of *N*_*s*_ = 4 stimulation sites and σ = 0.5. Stimulation sites are depicted by filled circles. **(B)** Stimulation signals ρ_*k*_(*t*)*P*(*t*), $k=\bar{1,\;4}$, see Equation (2), for the 4-site CR stimulation protocol are shown by the same color as the corresponding stimulation sites from plot **(A)**.

CR stimulation is delivered to the phase ensemble (Equation 1) in the following setup: The oscillators are assumed to be equidistantly arranged on a 1D lattice of length *L* = 10 with lattice coordinates *x*_*j*_ = (*j* − 1)*L*/(*N* − 1), $j=\bar{1,\;N}$. The stimulation signals are sequentially delivered via *N*_*s*_ stimulation sites, which are equidistantly spaced within the stimulated ensembles with coordinates $c_{k}=(k-\frac{1}{2})L/N_{s}$, $k=\bar{1,\;N_{s}}$. The control of system (Equation 1) by CR stimulation of strength *I* is modeled by the following stimulation term (Tass, 2003a,b) in Equation (1):

(2) $$S_{j}(t)=I\sum_{k=1}^{N_{s}}D(x_{j},\;k)\rho_{k}(t)P(t)\cos\theta_{j},$$

where *P*(*t*) is a HF pulse train of unit amplitude, and ρ_*k*_(*t*) are the indicator functions such that ρ_*k*_(*t*) = 1 if the *k*th stimulation site is active and ρ_*k*_(*t*) = 0 otherwise. Functions *D*(*x*_*j*_, *k*) define the spatial spread of the stimulation signals in the target population such that the impact of the stimulation signal delivered via the *k*th stimulating site on the *j*th oscillator depends on the distance |*x*_*j*_ − *c*_*k*_| between the oscillator and the stimulation site. Following Richardson et al. (2003) we consider a quadratic spatial profile of the current spread

(3) $$D(x_{j},\;k)=\frac{1}{1+{\left(x_{j}-c_{k}\right)}^{2}/\sigma^{2}},$$

where σ defines the spatial decay rate of the stimulation current (Figure 1A).

The CR stimulation signals are short HF pulse trains (i.e., bursts with an intra-burst frequency as for standard HF DBS) sequentially delivered via different stimulation sites (Tass, 2003a,b; Tass et al., 2012b). To define the stimulation signals in our models, we consider a long sequence of rectangular pulses of unit amplitude *P*(*t*) with the pulse period *T*_*p*_ = 0.025 (i.e., 40 pulses per time unit) and pulse width *T*_*p*_/2 = 0.0125. Then the stimulation signal delivered to the neurons via the *k*th stimulation site during its active period reads ρ_*k*_(*t*)*P*(*t*), where ρ_*k*_(*t*) controls the switching on and off of the *k*th stimulation site as defined above. During each stimulation cycle of length *T*, all *N*_*s*_ stimulation sites are sequentially activated delivering an HF pulse train of length *T*/*N*_*s*_, respectively (Figure 1B). For the phase oscillators (Equation 1) the length of the CR stimulation cycle is considered *T* = 2 which equals the mean period of the stimulation-free synchronized phase ensemble.

### 2.3. Spiking neuronal oscillators

In the previous section 2.1 we introduced the phase ensemble (Equation 1) controlled by CR stimulation. For applications, it is important to reveal whether the results for this model presented in sections 3.1 and 3.2 are robust and generic enough to be valid for more realistic neuronal models. Here we present a network of FHN (FitzHugh, 1961; Nagumo et al., 1962) spiking neurons interacting via excitatory chemical synapses,

(4) $$\begin{array}{l} \;{\dot{v}}_{j}=v_{j}-\frac{1}{3}v_{j}^{3}-w_{j}+1+I_{j}^{\left(\text{syn}\right)}+I_{j}^{\left(\text{stim}\right)},\\ {\dot{w}}_{j}=\varepsilon_{j}(v_{j}+0.7-0.8w_{j}),\\ \;{\dot{s}}_{j}=\frac{2(1-s_{j})}{1+\exp(-10v_{j})}-s_{j},\;j=1,\;2,\;\ldots ,\;N. \end{array}$$

The variable *v*_*j*_ models the membrane potential of a single neuron, *w*_*j*_ is a recovery variable, and the parameter ε_*j*_ determines the natural spiking frequency of a neuron (number of spikes per time unit). The synaptic coupling among the neurons is realized via post-synaptic potentials *s*_*j*_ triggered by spikes of neuron *j* (Terman, 2005; Izhikevich, 2007), which are modeled by additional differential equations for *s*_*j*_, see Terman et al. (2002) and Terman (2005). Then the synaptic current *I*^(syn)^_*j*_ in Equation (4) reads

(5) $$I_{j}^{\left(\text{syn}\right)}=C(V-v_{j})\frac{1}{N}\sum_{k=1}^{N}s_{k},$$

where *V* is a reversal potential, taken as *V* = 2 to realize an excitatory coupling, and *C* defines the coupling strength. We consider *N* = 400 neurons, coupling strength *C* = 0.11, and parameters ε_*j*_ are Gaussian distributed with mean 0.08 and standard deviation 0.002.

In order to quantify the extent of synchronization in this network, we use the order parameters *R*_*m*_, *m* ≥ 1, defined for the phase ensemble, see section 2.1, where phase θ_*j*_ of neuron *j* is approximated based on its spike timings *t*_*j*, *k*_: θ_*j*_(*t*) = 2π(*t* − *t*_*j*, *k*_)/(*t*_*j*, *k* + 1_ − *t*_*j*, *k*_) + 2π*k*, *t*_*j*, *k*_ ≤ *t* < *t*_*j*, *k* + 1_, *k* = 0, 1, 2,… (Pikovsky et al., 2001). For example, the time-averaged first order parameter 〈*R*_1_〉 ≈ 0.96 for the considered coupling strength and distribution of parameters ε_*j*_.

The stimulation current *I*^(stim)^_*j*_ in Equation (4) of CR stimulation is given by the following expression:

(6) $$I_{j}^{\left(\text{stim}\right)}=I\sum_{k=1}^{N_{s}}D(x_{j},\;k)\rho_{k}(t)P(t),$$

where the functions *D*, ρ_*k*_, and *P* are the same, as defined in section 2.2 for the ensemble of phase oscillators. We use the stimulation period *T* = 38 of CR stimulation, which approximates the period of the intrinsic spiking dynamics of the neuronal ensemble (Equations 4, 5). The period of the high-frequency pulse train *P*(*t*) is taken *T*_*p*_ = 0.5, and the pulse width *T*_*p*_/2 = 0.25.

### 2.4. Bursting neuronal oscillators

Synchronized firing of bursting neurons has been reported in basal ganglia of MPTP-treated monkeys (Bergman et al., 1994, 1998). In this section we present a model network of coupled adaptive exponential integrate-and-fire (aEIF) bursting neurons (Brette and Gerstner, 2005; Naud et al., 2008; Touboul and Brette, 2008). The model is given by the following equations for the membrane potentials *V*_*j*_ and adaptation currents *w*_*j*_:

(7) $$\begin{array}{l} C{\dot{V}}_{j}=-g_{L}(V_{j}-E_{L})+g_{L}\Delta_{T}\exp\left(\frac{V_{j}-V_{T}}{\Delta_{T}}\right)-w_{j}+I_{j}^{\left(\text{syn}\right)}+I_{j}^{\left(\text{stim}\right)}+I_{j},\\ \;{\dot{w}}_{j}=(a(V_{j}-E_{L})-w_{j})/\tau_{w},\;j=1,\;2,\;\ldots ,\;N. \end{array}$$

The *j*th oscillator emits a spike if its membrane potential *V*_*j*_ overcomes some threshold, here we set it to −25 (mV). At this moment the variables (*V*_*j*_, *w*_*j*_) are instantaneously reset to the values:

$$\begin{array}{l} \;V_{j}\to V_{r},\\ w_{j}\to w_{j}+b. \end{array}$$

The parameters of the model are *g*_*L*_ = 30 nS, *E*_*L*_ = −70.6 mV, *V*_*T*_ = −50.4 mV, Δ_*T*_ = 2 mV, τ_*w*_ = 40 ms, *a* = 4 nS, *b* = 80 pA, *V*_*r*_ = −47.2 mV, and *C* = 281 pF. The values *I*_*j*_ are randomly chosen according to a Gaussian distribution with mean 780 and σ_*I*_ = 1.0 pA. These parameters imply a bursting mode of the oscillators (Touboul and Brette, 2008) with period ≈ 70 ms. The synaptic coupling *I*^(syn)^_*j*_ is given by

$$\begin{array}{l} I_{j}^{\left(\text{syn}\right)}=K(V_{\text{r}.\text{p}.}-V_{j})\frac{1}{N}\sum_{k=1}^{N}\alpha (t-t_{k}^{\left(\text{sp}\right)}),\\ \alpha (x)=4x\;\exp(-4x), \end{array}$$

where *K* is the coupling strength, *t*^(sp)^_*k*_ is the last spike of the *k*th oscillator, and *V*_r.p._ = −20 mV is a reversal potential. We consider *K* = 12 nS such that the time-averaged first order parameter 〈*R*_1_〉 ≈ 0.92 calculated as for the FHN ensemble except that the timings *t*_*j*, *k*_ stand for the onsets of bursts. The stimulation current *I*^(stim)^_*j*_ of CR stimulation is considered in the form (Equation 6) with parameter *T*_*p*_ = 2 ms. The stimulation period *T* = 70 ms, and the number of neurons in the ensemble is taken *N* = 200.

## 3. Results

### 3.1. Effects of continuous CR stimulation of phase oscillators

The desynchronizing impact of CR stimulation on the controlled oscillators depends on the stimulation parameters mentioned above, see Lysyansky et al. (2011) where, in particular, the role of the stimulation strength *I* and the current decay rate σ was investigated. In the present work the main attention is paid to the impact of the number of stimulating sites *N*_*s*_ on desynchronizing effect. CR stimulation can be administered either continuously, where the stimulation cycles described above of length *T* are applied one after another (without interruption), or in an intermittent way, where a few stimulation cycles are repeatedly followed by a few cycles without stimulation. In this section we consider the former stimulation protocol.

**Figure 2:**
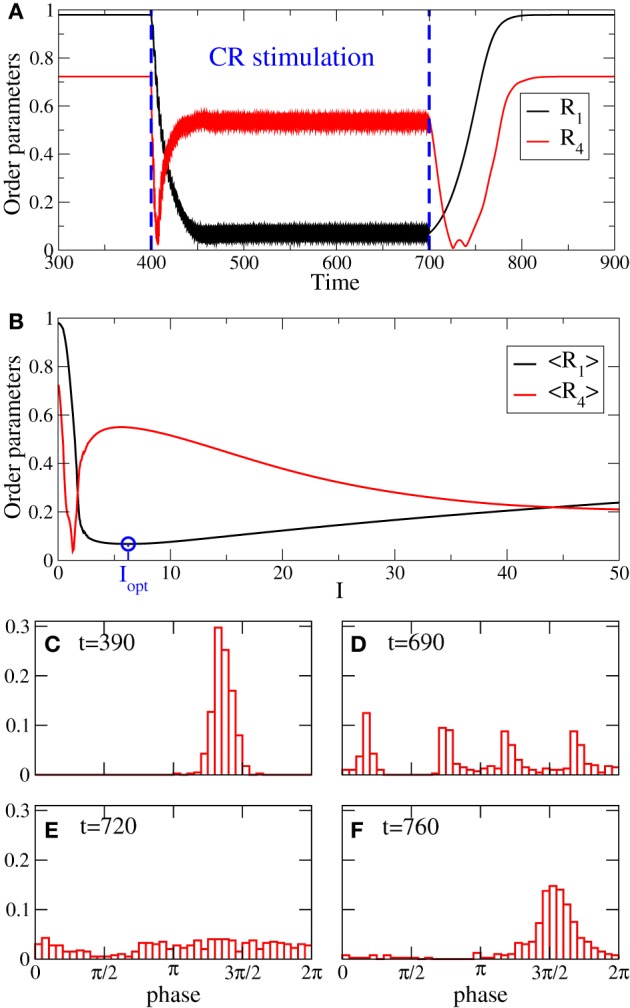
**(A)** Time courses of the order parameters *R*_1_ and *R*_4_ for the phase oscillators (Equation 1) controlled by 4-site CR stimulation (Equation 2) administered to synchronized oscillators in the time interval *t* ∈ [400, 700]. **(B)** Time-averaged (over 400 stimulation periods *T*) order parameters 〈*R*_1_〉 and 〈*R*_4_〉 vs. stimulation strength *I*. Blue circle depicts the minimal value of 〈*R*_1_〉 achieved for the optimal stimulation strength *I*_opt_ = 6.25, which is taken for the simulation in plot **(A)**. **(C–F)** Snapshots of the phase distribution density histograms at the times indicated in the plots. Current decay rate σ = 0.5 in Equation (2).

Examples of the time courses of the order parameters *R*_1_ and *R*_4_ before, during, and after 4-site continuous CR stimulation are illustrated in Figure 2A. CR stimulation is administered to an ensemble of strongly synchronized oscillators (Equation 1) (switched on at *t* = 400) where, because of the strong enough coupling, the phases are narrowly distributed (Figure 2C), and the first order parameter attains a large value *R*_1_ ≈ 0.98 in the pre-stimulus interval (Figure 2A for *t* < 400). After a short transient the stimulation suppresses the in-phase synchronization marked by small values of *R*_1_ (Figure 2A, black curve for 400 < *t* < 700). We found that during the stimulation the order parameters slightly fluctuate around their mean values 〈*R*_1_〉 ≈ 0.07, 〈*R*_2_〉 ≈ 0.13, 〈*R*_3_〉 ≈ 0.17, and 〈*R*_4_〉 ≈ 0.55 (Figure 2A, only *R*_1_ and *R*_4_ are depicted by black and red curves, respectively). Therefore, continuous CR stimulation administered via 4 stimulation sites can reliably induce a 4-cluster state such that the phases are grouped into four clusters equidistantly spaced on the unit circle (Figure 2D). The emergence of the cluster state is reflected by large values of *R*_4_ combined with small values of *R*_1_, *R*_2_, and *R*_3_, see section 2.1 and Lysyansky et al. (2011). After the stimulation is switched off (at *t* = 700) the oscillatory ensemble transiently relaxes from the stimulation-induced cluster state to a desynchronized state where the phases get nearly uniformly distributed (Figure 2E), and which is characterized by small values of both order parameters *R*_1_ and *R*_4_ (Figure 2A). Then the desynchronization is followed by a resynchronization due to the persistent strong coupling. The phases of the stimulation-free oscillators form a single cluster (Figure 2F) and approach the original strongly synchronized state (Figure 2C), and the order parameters increase (Figure 2A for *t* > 800). The discussed cluster state induced by the continuous CR stimulation is a robust phenomenon (Lysyansky et al., 2011). For example, for the considered 4-site CR stimulation there exists a range of the stimulation strength *I* where the time-averaged first and fourth order parameters 〈*R*_1_〉 and 〈*R*_4_〉 attain small and large values, respectively, which is characteristic for a four-cluster state, see Figure 2B. The minimal value of 〈*R*_1_〉 obtained for the optimal stimulation strength *I*_opt_ is depicted by the blue circle in Figure 2B.

**Figure 3:**
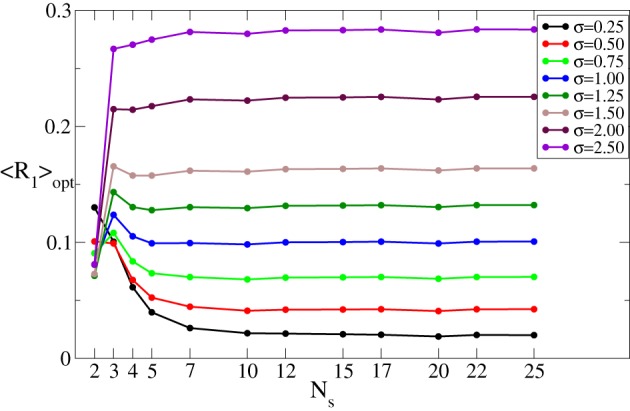
**Minimal values 〈*R*_1_〉_opt_ of the time-averaged first order parameter 〈*R*_1_〉 of the phase ensemble (Equation 1) controlled by continuous CR stimulation vs. the number of stimulation sites *N*_*s*_ for different values of the parameter σ in Equation (3) as indicated in the legend**. The stimulation strength *I* was varied in the interval [0, 60], i.e., *I*_max_ = 60. Other parameters as in Figure 2.

As mentioned in the Introduction, in this study we estimate the desynchronizing impact of CR stimulation for different numbers of stimulation sites *N*_*s*_. For this the stimulation strength *I* is varied in some interval [0, *I*_max_] whereas the other parameters remain fixed. The minimal (optimal) value 〈*R*_1_〉_opt_ of the time-averaged first order parameter 〈*R*_1_〉 achieved in the considered interval of *I* (see Figure 2B) is then used to quantify the optimal effect of CR stimulation for a given number of stimulation sites *N*_*s*_. As *N*_*s*_ increases, the optimal values 〈*R*_1_〉_opt_ may demonstrate different behaviors depending on the spatial decay rate σ of the stimulation current, see Figure 3. 〈*R*_1_〉_opt_ saturates for large *N*_*s*_, and additional stimulation sites do not further improve the optimal effect of CR stimulation. The saturation levels of 〈*R*_1_〉_opt_ decisively depend on the values of σ, where small (large) σ implies small (large) values of 〈*R*_1_〉_opt_. Moreover, small σ (≲0.5) ensures that the increasing number of stimulation sites *N*_*s*_ up to ≈5–10 sites improves the stimulation outcome as compared to the stimulation delivered via *N*_*s*_ ≈2–3 sites. In contrast, large σ (≳1.25) implies that *N*_*s*_ = 2 sites is the most preferable choice, and larger *N*_*s*_ leads to larger values of 〈*R*_1_〉_opt_ and to a suboptimal desynchronizing effect of continuous CR stimulation. For intermediate values of σ (≈0.75–1.00) 〈*R*_1_〉_opt_ does not significantly change with varying number of stimulation sites.

In summary, if the properties of the neuronal tissue imply a spatially selective stimulation profile, corresponding to a small σ, additional stimulation sites can improve the desynchronizing effect of CR stimulation. In contrast, a small number of stimulation sites is the best choice for a broad profile of the spatial current decay (i.e., for large σ).

### 3.2. Effects of intermittent CR stimulation of phase oscillators

The suggested protocol of CR stimulation (Tass, 2003a,b) implies an intermittent (ON–OFF) administration of the stimulation, where several cycles of CR stimulation (ON-cycles) are followed by a few rest cycles without stimulation (OFF-cycles). Such an intermittent stimulation allows the oscillators to freely evolve during the OFF-cycles according to their intrinsic unperturbed dynamics. During the post-stimulus transient (OFF cycles) the oscillatory ensemble (Equation 1) relaxes into a desynchronized state followed by resynchronization, both phenomena (transient desynchronization and subsequent resynchronization) being a consequence of the persisting strong coupling in the network (Tass, 2003a,b). This effect is illustrated in Figure 2A, where the post-stimulus desynchronization is reflected by small values of the order parameters. Intermittent stimulation can also decrease the amount of current delivered to the target tissue. This, in turn, can minimize the spread of the stimulation to the neighboring areas and, hence, prevent from possible side effects. In networks with spike timing-dependent plasticity (STDP) (Markram et al., 1997; Bi and Poo, 1998) intermittent CR stimulation causes an anti-kindling effect (Tass and Majtanik, 2006; Hauptmann and Tass, 2007). Anti-kindling is a desynchronization-induced unlearning of pathologically upregulated synaptic connectivity and, in turn, synchrony, i.e., a stabilization of a desynchronized activity in a network, which persists even if the stimulation is completely switched off (Tass and Majtanik, 2006; Hauptmann and Tass, 2009). The time scale of STDP is essentially larger than the *m* : *n* timing of the intermittent stimulation and has an order of tens or hundreds periods of the oscillation (Bi and Poo, 1998; Hauptmann and Tass, 2009). In this paper we investigate the impact of CR stimulation on oscillatory networks without STDP.

**Figure 4:**
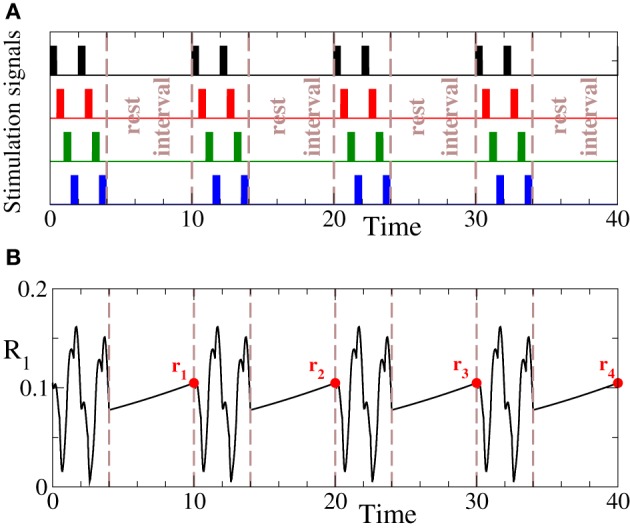
**(A)** Schematic illustration of the intermittent *m* : *n* ON–OFF CR stimulation with *m* = 2 and *n* = 3 stimulation and rest cycles of length *T*, respectively. Number of stimulation sites *N*_*s*_ = 4. **(B)** Dynamics of the first order parameter *R*_1_ for the phase ensemble (Equation 1) subjected to the intermittent CR stimulation. Red circles depict the maximal values *r*_*k*_ of the order parameter achieved in the corresponding rest intervals ℐ_*k*_. Stimulation strength *I* = 10 and σ = 0.5. Other parameters as in Figure 2.

To quantify the desynchronizing effect of intermittent CR stimulation, we use an approach suggested in Lysyansky et al. (2011). In each rest interval ℐ_*k*_ we evaluate the maximal value of the first order parameter such that a set of the maximal values $r_{k}=\underset{t\in {ℐ}_{k}}{\max}R_{1}(t),\;k=1,\;2,\;\ldots$, *k* = 1, 2,… is calculated. For example, during the rest intervals the first order parameter *R*_1_(*t*) can increase and reach its maximal values *r*_*k*_ at the end of the OFF cycles (Figure 4B, red circles). The mean value of *r*_*k*_, i.e., $\langle r\rangle =N_{\text{rp}}^{-1}\sum_{k=1}^{N_{\text{rp}}}r_{k}$, where *N*_rp_ is the number of rest periods, is then used to estimate the effect of the intermittent ON–OFF CR stimulation. In what follows we denote by *m* and *n* the number of the ON and OFF cycles of length *T* of the interchanging stimulation and rest time intervals, respectively, where *T* is the stimulation period (Figure 4A). If *m* and *n* can attain non-integer values, an interesting anti-resonance-like behavior of 〈*r*〉 vs. *m* and *n* can be observed (Lysyansky et al., 2011). In the present paper we consider integer values of *m* and *n* only and analyze the impact of the number of stimulation sites *N*_*s*_ on the optimal value of 〈*r*〉. We also vary the stimulation strength *I* as well as the stimulation timing, i.e., the values of *m* and *n* in order to find optimal parameters for the intermittent CR stimulation.

**Figure 5:**
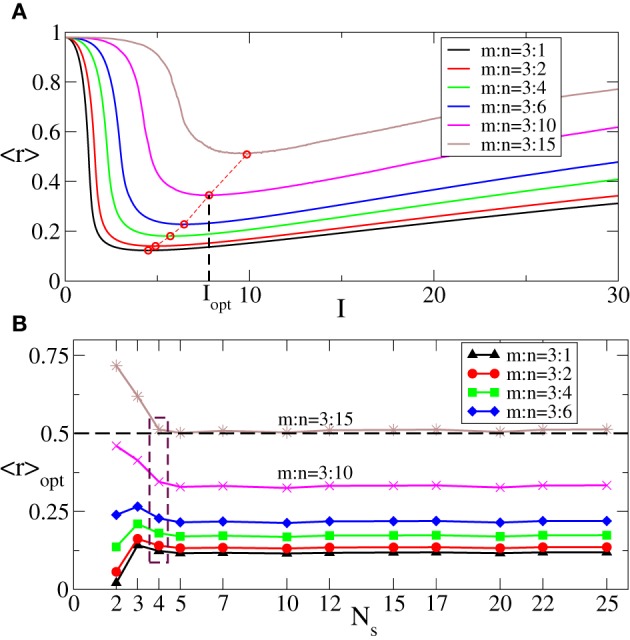
**(A)** Behavior of the order parameter 〈*r*〉 [averaged maxima of *R*_1_(*t*) of the phase ensemble (Equation 1) during the rest intervals, see text for definition] vs. the stimulation strength *I* of the intermittent CR stimulation for fixed *m* = 3 and different numbers *n* of the stimulation and rest cycles of length *T*, as indicated in the legend. Red circles indicate the minimal values 〈*r*〉_opt_ of the order parameter 〈*r*〉 obtained for the corresponding optimal stimulation intensities *I*_opt_. **(B)** 〈*r*〉_opt_ vs. the number of stimulation sites *N*_*s*_ for different *n* indicated in the legend. The dashed horizontal line depicts the threshold 〈*r*〉_opt_ = 0.5, and the dashed box indicates the number of sites *N*_*s*_ = 4 used in plot **(A)**. σ = 1.0, the number of the rest periods used for calculations *N*_rp_ = 400, and other parameters as in Figure 3.

In Figure 5A we illustrate the dynamics of 〈*r*〉 vs. the stimulation intensity *I* for fixed number *m* = 3 of the ON cycles and different numbers *n* of the OFF cycles. As in the case of the continuous stimulation protocol (Figure 2B), there exists an optimal stimulation strength *I*_opt_ = *I*_opt_(*m*, *n*), where 〈*r*〉 attains a minimal value 〈*r*〉_opt_ (Figure 5A, red circles). Increasing *n* results in larger values of 〈*r*〉 and 〈*r*〉_opt_ since longer rest intervals imply more time for resynchronization and, hence, larger maximal values of *R*_1_ occurring during the rest intervals. We follow the optimal values 〈*r*〉_opt_ as the number of stimulation sites *N*_*s*_ increases and find that 〈*r*〉_opt_ saturates if *N*_*s*_ gets large enough (Figure 5B). This phenomenon is tightly related to the saturation observed for the continuous CR stimulation (Figure 3).

**Figure 6:**
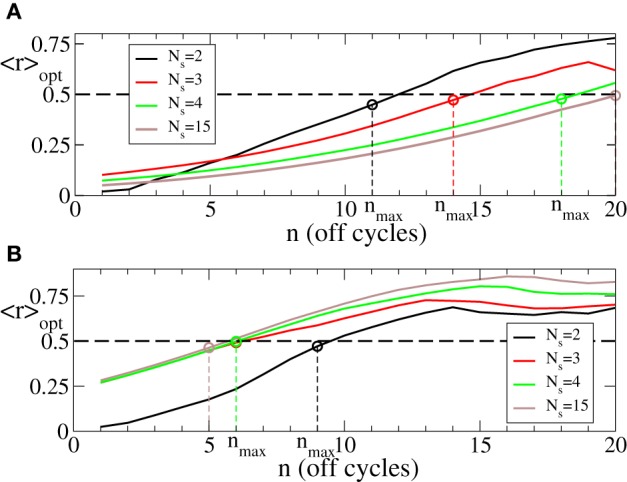
**Behavior of 〈*r*〉_opt_ of the phase ensemble (Equation 1) stimulated by the intermittent CR stimulation vs. the number *n* of OFF cycles in the rest intervals for (A) σ = 0.5 and (B) σ = 2.0 in Equation (3) and for different numbers of stimulation sites *N*_*s*_ as indicated in the legends**. Empty circles indicate the maximal admissible length *n*_max_ of the rest interval for a given *N*_*s*_ for which 〈*r*〉_opt_ still remains below or equal to 0.5. Number of the ON cycles *m* = 3, and other parameters as in Figure 3.

Since we are interested in the longest possible post-stimulus desynchronized transient as discussed above, we are looking for the stimulation parameters leading to the maximal admissible length *n*_max_ of the rest intervals. For this we first investigate the dynamics of 〈*r*〉_opt_ induced by the intermittent CR stimulation vs. the length of the rest intervals *n* for different numbers of stimulation sites *N*_*s*_, see Figure 6. As expected (see also Figure 5A), 〈*r*〉_opt_ = 〈*r*〉_opt_(*n*) increases as the rest intervals get longer. At some *n* = *n*_max_ + 1, 〈*r*〉_opt_ exceeds the predefined threshold 〈*r*〉_opt_ = 0.5 (Figure 6, dashed line). We thus consider the value *n* = *n*_max_ (Figure 6, empty circles) as the maximal admissible length of the rest intervals for the stimulation strength *I* ∈ [0, *I*_max_]. *n*_max_ can thus serve as a criterion for an optimal parameter choice for the intermittent CR stimulation since large *n*_max_ guarantees long stimulation-free OFF intervals. We found that an increasing number of stimulation sites *N*_*s*_ can prolong the admissible rest periods, i.e., *n*_max_ increases, and thus improve the desynchronizing impact of the intermittent CR stimulation if the latter is spatially rather selective, i.e., the stimulation current is narrowly distributed around the stimulation site within the stimulated population (Figure 6A for σ = 0.5). For a broad spatial spread of the stimulation current the situation, however, is exactly opposite, i.e., *n*_max_ decreases for larger *N*_*s*_ (Figure 6B for σ = 2.0) such that the longest rest periods can be achieved for the smallest number of stimulation sites *N*_*s*_ = 2.

**Figure 7:**
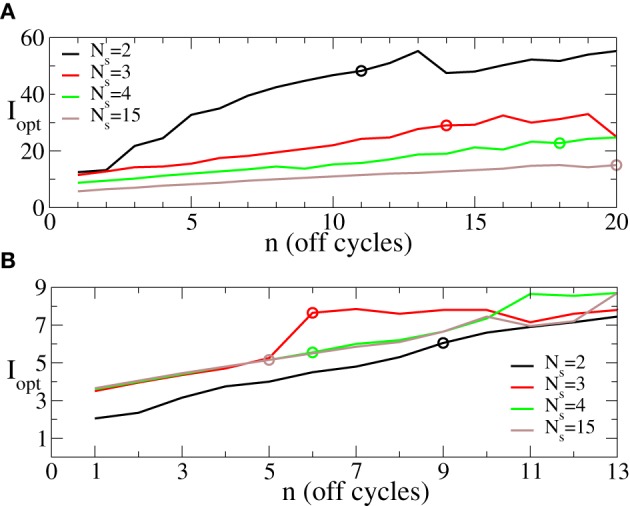
**Behavior of the optimal stimulation strength *I*_opt_ ∈ [0, 60] providing the minimal values 〈*r*〉_opt_ shown in Figure 6 vs. the number *n* of OFF cycles in the rest intervals for (A) σ = 0.5 and (B) σ = 2.0 in Equation (3) and for different numbers of the stimulation sites *N*_*s*_ as indicated in the legend**. Empty circles indicate the optimal stimulation strength *I*_opt_ at which the maximal admissible length *n*_max_ of the rest interval can be achieved for a given *N*_*s*_. Other parameters as in Figure 6.

The minimal values 〈*r*〉_opt_ of the first order parameter illustrated in Figure 6 are attained at the optimal stimulation strengths *I* = *I*_opt_ ∈ [0, *I*_max_] (Figure 5A) shown in Figure 7 vs. the number *n* of the OFF cycles in the rest intervals. Accordingly, one has to increase the stimulation strength in order to reach the possible minimal value of the order parameter 〈*r*〉_opt_ during the longer post-stimulus rest periods if the number of stimulation sites *N*_*s*_ is fixed. Nevertheless, the optimal intermittent CR stimulation with the maximal admissible length *n*_max_ of the rest periods (Figure 6) can be realized for smaller optimal stimulation strength *I*_opt_ if the number of stimulation sites is increased (Figure 7, empty circles). Note, this effect is well pronounced for small σ (Figure 7A) in contrast to the case of large σ (Figure 7B).

**Figure 8:**
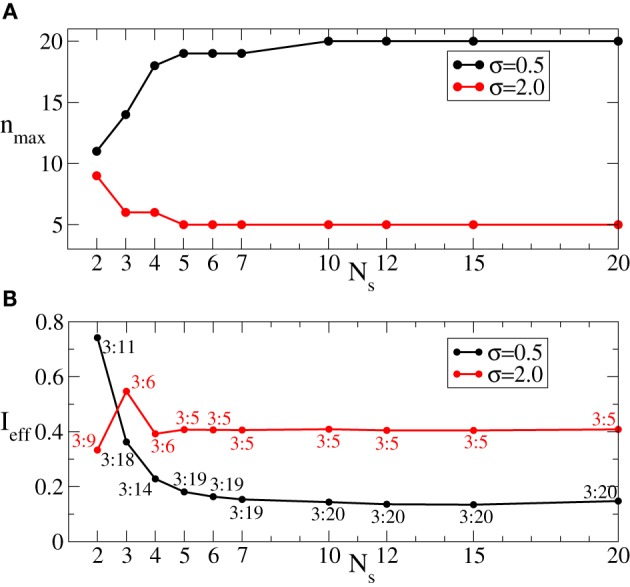
**(A)** Maximal admissible number *n*_max_ of OFF cycles and **(B)** effective amount of the stimulation *I*_eff_ received on average by a single oscillator of the phase ensemble (Equation 1) during the intermittent CR stimulation vs. the number of stimulation sites *N*_*s*_ for σ = 0.5 (black curves) and σ = 2 (red curves). Other parameters as in Figure 6.

We have shown above that the desynchronizing impact of the continuous as well as the intermittent CR stimulation essentially depends on the spatial decay rate of the stimulation current σ as the number of stimulation sites varies (Figures 3, 6). We summarize our findings in Figure 8. If the number of stimulation sites *N*_*s*_ increases, the maximal length *n*_max_ of the admissible rest periods can either increase or decrease depending on the values of σ, see Figure 8A. The spatially selective stimulation with small σ (Figure 8A, black curve for σ = 0.5) implies relatively short rest intervals for the stimulation delivered via small number of sites. Additional stimulation sites strongly elongate the admissible rest intervals, which, however, saturate for large *N*_*s*_. In contrast, the intermittent CR stimulation with a broad spatial spread of the stimulation signal (large σ) admits the longest rest periods for a small number of stimulation sites (Figure 8A, red curve for σ = 2). Adding further stimulation sites can only worsen the stimulation outcome in this case.

As follows from Figures 5A, 7 the optimal value 〈*r*〉_opt_ is attained for larger optimal stimulation strength *I*_opt_ if the number *n* of the OFF cycles increases. It is thus important to clarify how much stimulation an individual oscillator receives for the optimal intermittent CR stimulation as in Figure 8A. We therefore calculate the effective amount of the administered stimulation $I_{\text{eff}}=0.5I\frac{m}{m+n}\frac{1}{N_{s}\times N}\sum_{k=1}^{N_{s}}\sum_{j=1}^{N}D(x_{j},\;k)$ which is the time and ensemble average of the stimulation signal (Equation 2). In Figure 8B the values of *I*_eff_ are plotted vs. the number of stimulation sites *N*_*s*_ for the maximal admissible length of the rest interval *n*_max_ from Figure 8A. For small σ (spatially selective stimulation) the desynchronizing effect of the intermittent CR stimulation is significantly improved by increasing *N*_*s*_, where the length of the admissible rest periods is increased (Figure 8A, black curve), and the amount of required stimulation is decreased (Figure 8B, black curve). For the large σ the situation is opposite, see Figures 8A,B (red curves). However, due to the saturation effect, the improvement of the stimulation impact for the considered small σ = 0.5 becomes less effective if the number of stimulation sites gets larger than *N*_*s*_ ≈ 5 − 7 sites (Figures 8A,B, black curves). It is thus unreasonable to strongly increase the number of stimulation sites, which can either induce no significant improvements of the desynchronizing impact of CR stimulation, or even worsen it if the spatial stimulation profile is broad enough.

**Figure 9:**
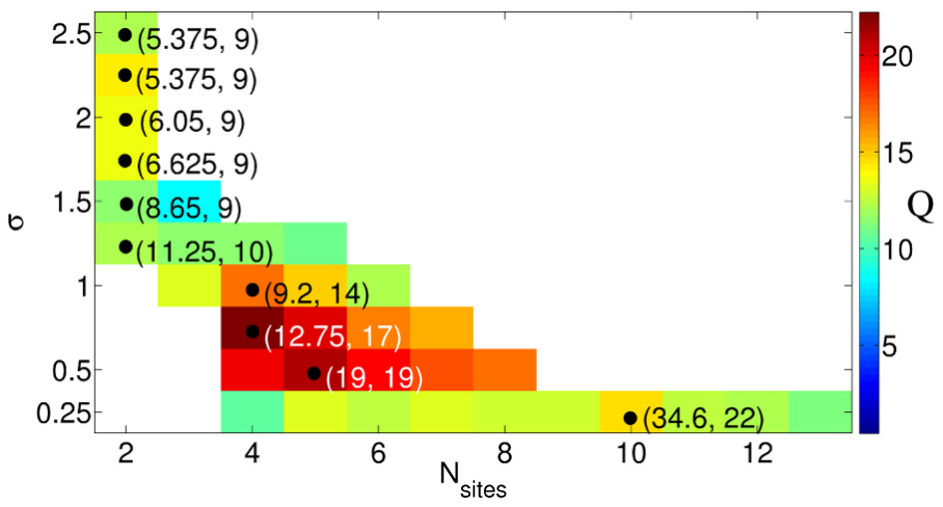
**Quality ***Q*** = ***n*_max_**/(***I*_eff_** × ***N*_*s*_**) of the intermittent CR stimulation depicted in color ranging from blue (small values) to red (large values), see the color bar**. The optimal numbers of stimulation sites *N*_*s*, opt_, where the maximal values $Q_{\text{max}}(\sigma )=\underset{N_{s}}{\max}Q(N_{s},\;\sigma )$ are achieved for fixed σ, are indicated by black circles. The corresponding parameters (*I*_opt_, n_max_) of the stimulation are given in the round brackets. The values of *Q* smaller than 0.7*Q*_max_ are not shown (white regions). Other parameters as in Figure 6.

Since the best intermittent CR stimulation is leading to the longest OFF periods achieved for a small stimulation current, we introduce a measure *Q* of the quality of the intermittent CR stimulation, which is proportional to the maximal admissible length of the rest intervals given by *n*_max_ and inversely proportional to the effective amount of the stimulation *I*_eff_ and the number of stimulation sites *N*_*s*_, *Q* = *n*_max_/(*I*_eff_ × *N*_*s*_). This function estimates the efficacy of CR stimulation “per each stimulation site” such that for similar values of *n*_max_/*I*_eff_ more preferable is the stimulation setup with a smaller number of sites. We found that for fixed spatial decay rate of the stimulation signal from a range σ ∈ [0.25, 2.5], *Q* achieves a clear maximum *Q*_max_ = *Q*_max_(σ) for an optimal numbers of stimulation sites *N*_*s*, opt_ depicted by a black circle in Figure 9, respectively. Ranges of *N*_*s*_ are indicated by colored stripes where the quality *Q* of the stimulation does not deviate for more than 30% from its best value *Q*_max_. In contrast, the white regions correspond to parameter values of clearly sub-optimal CR stimulation and should be avoided. This diagram supports our conclusion that an optimal CR stimulation has to be administered via a small number of sites if the stimulation current is broadly spread in the tissue, i.e., if σ is large, whereas for small σ (spatially selective stimulation) a larger number of stimulation sites leads to a better desynchronizing impact of CR stimulation.

### 3.3. Results for spiking neuronal model

**Figure 10:**
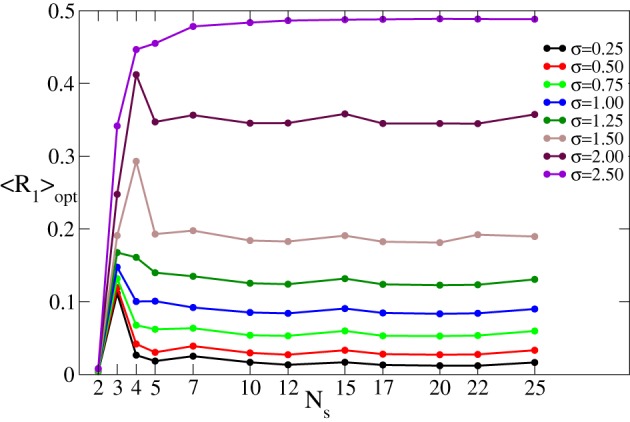
**Minimal values 〈*R*_1_〉_opt_ of the time-averaged first order parameter 〈*R*_1_〉 of the FHN neuronal ensemble (Equations 4–6) controlled by continuous CR stimulation vs. the number of stimulation sites *N*_*s*_ for different values of the parameter σ in Equation (3) as indicated in the legend**. The stimulation strength *I* has been varied in the interval [0, 10].

For the FHN model (Equations 4, 5) controlled by CR stimulation (Equation 6) we use the same evaluation techniques applied to the phase oscillators (Equation 1) and described in section 3.1. We thus calculate the optimal (minimal) values 〈*R*_1_〉_opt_ of the time-averaged first order parameter 〈*R*_1_〉 obtained under variation of the stimulation strength *I* ∈ [0, 10]. The effect of continuous CR stimulation on the FHN network is illustrated in Figure 10 vs. the number of stimulation sites *N*_*s*_. Different curves in the plot represent the stimulation-induced minimal values of the first order parameter 〈*R*_1_〉_opt_ for different values of the spatial decay rate σ. This diagram is very similar to that in Figure 3 obtained for the phase oscillators. Indeed, 〈*R*_1_〉_opt_ saturates for large number of stimulation sites. The saturation levels of 〈*R*_1_〉_opt_ depend on the values of σ as for the phase oscillators, such that for small (large) σ CR stimulation leads to a small (large) order parameter which saturates for large *N*_*s*_.

**Figure 11:**
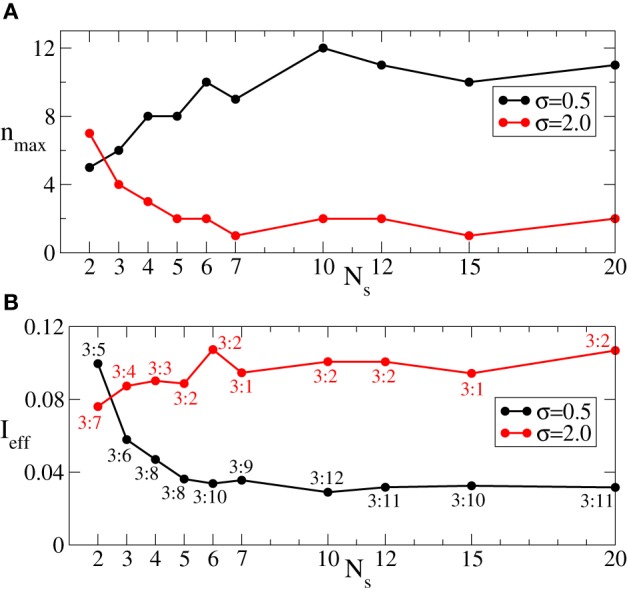
**(A)** Maximal admissible number *n*_max_ of OFF cycles and **(B)** effective amount of the stimulation *I*_eff_ received on average by a single neuron of the FHN ensemble (Equations 4–6) during the intermittent CR stimulation vs. the number of stimulation sites *N*_*s*_ for σ = 0.5 (black curves) and σ = 2 (red curves). Number of ON cycles *m* = 3, and other parameters as in Figure 10.

Also for the intermittent ON–OFF CR stimulation of the FHN ensemble the impact of the stimulation can be quantified in the same way as described in section 3.2 for the network of phase oscillators. In particular, Figure 11A illustrates the influence of the number of stimulation sites *N*_*s*_ on the maximal admissible length *n*_max_ of the rest intervals. As for the phase oscillators (Figure 8A), a spatially selective (i.e., with small σ) intermittent CR stimulation is less effective for small number of stimulation sites *N*_*s*_ (*n*_max_ is small), whereas the length of the rest intervals can significantly be prolonged if *N*_*s*_ increases (Figure 11A, black curve for σ = 0.5). Simultaneously, the amount of the stimulation current *I*_eff_ received on average by a single neuron in the network decays with increasing *N*_*s*_ (Figure 11B, black curve). Therefore, a selective intermittent CR stimulation optimally delivered to a neuronal network via a large number of stimulation sites enables long stimulation-free desynchronization time intervals with a minimal amount of the administered stimulation current. In contrast, for a broad spatial spread of the stimulation current in the neuronal tissue (large σ) the intermittent CR stimulation has to be optimally administered via a small number on stimulation sites, e.g., *N*_*s*_ = 2. Only for such a stimulation protocol CR stimulation can lead to the longest possible rest periods achieved for the smallest possible delivered stimulation current (Figure 11, red curves for σ = 2). In this case, a larger number of stimulation sites can worsen the desired desynchronizing impact of CR stimulation.

Note, that for the FHN neuronal ensemble (Equations 4, 5) controlled by the intermittent CR stimulation (Equation 6) we have observed the same behavior of the order parameter 〈*r*〉_opt_ and the optimal stimulation strength *I*_opt_ vs. the number of the OFF cycles *n* in the rest intervals (figures are not shown) as for the phase ensemble, see Figures 6, 7. Together with the results illustrated in the above Figures 10, 11 this indicates the robustness of the reported phenomena, which can equally be found for phase oscillators and for spiking neurons interacting via excitatory synapses.

### 3.4. Results for the bursting neuronal model

**Figure 12:**
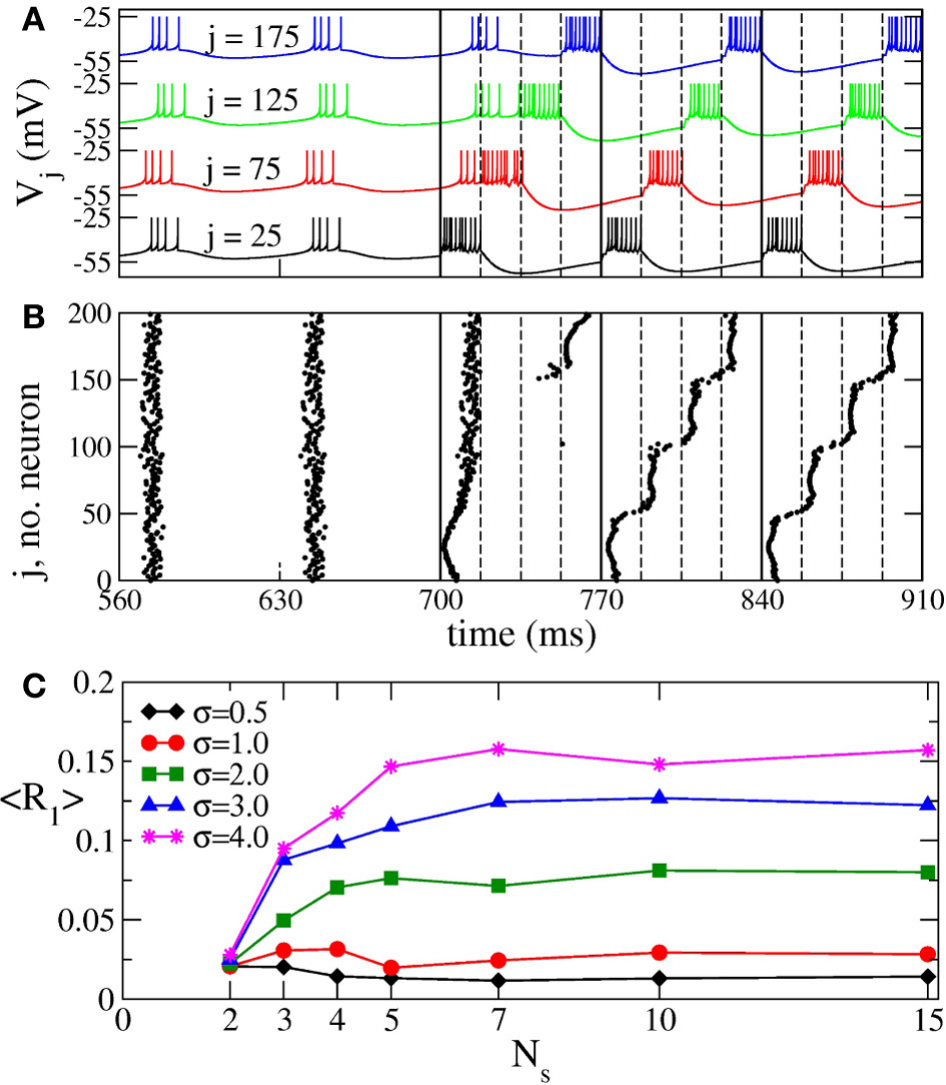
**Impact of continuous CR stimulation on the ensemble of aEIF bursting neurons (Equation 7)**. **(A)** Time courses of the membrane potentials *V*_*j*_ of four selected neurons with indices *j* indicated in the plot. The neurons are assigned to each of the four stimulation sites located at the same lattice coordinates. Stimulation begins at *t* = 700 ms with stimulation intensity *I* = 1550 pA, and spatial current decay rate σ = 0.5. The vertical solid lines comprise the CR stimulation cycles of length *T* = 70 ms, and the dashed lines indicate the time intervals, where the corresponding stimulation site is active. **(B)** The corresponding raster plot of the burst onsets. **(C)** Minimal values 〈*R*_1_〉_opt_ of the time-averaged first order parameter of the neuronal ensemble controlled by continuous CR stimulation vs. the number of stimulation sites *N*_*s*_ for different values of parameter σ in Equation (3) as indicated in the legend. The stimulation strength *I* was varied in the interval [0, 2000] pA.

When continuous CR stimulation is administered to synchronized aEIF bursting neurons (Equation 7), the in-phase synchronization is replaced by a cluster state (Figures 12A,B). The bursting neuronal discharges get organized in a few sub-groups (clusters) corresponding to the number of stimulation sites, and the neurons within the same cluster fire simultaneously with equidistant time shift between neighboring clusters. The same dynamics is observed for the phase oscillators (Figure 2) and FHN neurons. The synchronization order parameter of the aEIF neuronal ensemble is suppressed from 〈*R*_1_〉 ≈ 0.92 in the in-phase synchronized regime to 〈*R*_1_〉 ≈ 0.014 in the cluster state for the parameter values used in Figures 12A,B. In the latter regime the other order parameters are 〈*R*_2_〉 ≈ 0.063, 〈*R*_3_〉 ≈ 0.088, and 〈*R*_4_〉 ≈ 0.766, which is a clear indication of the 4-cluster state that can be seen in Figures 12A,B.

The spatial decay rate σ of the stimulation current and the number *N*_*s*_ of stimulation sites can significantly influence the desynchronizing impact of permanent CR stimulation as measured by the values of the first order parameter. In particular, for large σ the time-averaged first order parameter 〈*R*_1_〉 grows as *N*_*s*_ increases, whereas the opposite is true for small σ, see Figure 12C. This effect revealed for the considered bursting neurons is again in perfect agreement with the results obtained for the phase oscillators (Figure 3) and FHN spiking neurons (Figure 10).

**Figure 13:**
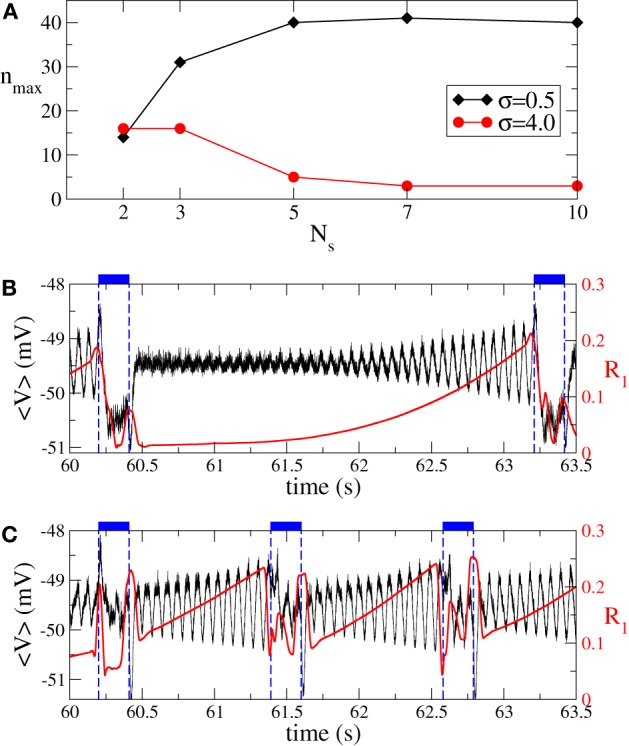
**Impact of intermittent ON–OFF CR stimulation on the ensemble of aEIF bursting neurons (Equation 7). (A)** Maximal admissible number *n*_max_ of OFF cycles vs. the number of stimulation sites *N*_*s*_ for σ = 0.5 (black diamonds) and σ = 4 (red circles). The synchronization threshold is considered 〈*r*〉_opt_ = 0.25, see section 3.2 for definition. **(B)** Time courses of the ensemble mean filed $\langle V\rangle =N^{-1}\sum_{j=1}^{N}V_{j}$ (black curve) and the first order parameter *R*_1_ (red curve, scale on the right vertical axis) between two successive ON epochs (indicated by dashed vertical lines and bars on the top of the plot). Parameters σ = 0.5, *n* = *n*_max_ = 40, *I* = *I*_opt_ = 392 pA, and *N*_*s*_ = 5. **(C)** The same as in **(B)** for the parameters σ = 0.5, *n* = *n*_max_ = 14, *I* = *I*_opt_ = 460 pA, and *N*_*s*_ = 2. Number of ON cycles *m* = 3, and other parameters as in Figure 12.

Also the intermittent ON–OFF CR stimulation of the aEIF neuronal ensemble can become more effective for a large number of stimulation sites. However, this requires spatially selective stimulation, i.e., small σ (Figure 13A, black diamonds). In this case the maximally admissible number of OFF intervals *n*_max_ increases with the number of stimulation sites, which permits longer time intervals with desynchronized dynamics of the stimulation-free neurons. We illustrate this effect on the synchronized dynamics for σ = 0.5 and different numbers of stimulation sites in Figures 13B,C. The suboptimal number of stimulation sites *N*_*s*_ = 2 (Figure 13C) implies shorter OFF-intervals and a faster re-increase of both order parameter *R*_1_ and amplitude of the ensemble mean field 〈*V*〉 during the OFF-intervals in comparison to the stimulation delivered via a larger number *N*_*s*_ = 5 of stimulation sites (Figure 13B). The situation is opposite for a broad spatial spread of the stimulation current in the neuronal tissue (large σ), where the intermittent CR stimulation can optimally be administered via a small number of stimulation sites only (Figure 13A, red circles). In this case, a larger number of stimulation sites can significantly shorten the admissible length of the rest intervals. The above effects of the intermittent CR stimulation for the aEIF bursting neurons (Equation 7) are again in good agreement with those observed for the phase oscillators (Figure 8) and FHN spiking neurons (Figure 11), which further confirms the generality of the presented results.

## 4. Discussion

An effective desynchronization of pathological neuronal synchronization, present in several neurological disorders like Parkinson's disease or essential tremor, is an important clinical challenge (Tass, 1999). The considered CR stimulation technique provides an effective means for the desynchronization of neuronal networks (Tass, 2003b). The problem of an appropriate calibration of the stimulation parameters like stimulation strength or timing parameters of the intermittent CR stimulation has been addressed in our previous study (Lysyansky et al., 2011). However, the impact of the number of stimulation sites has not been studied so far. This problem is computationally addressed in the present paper.

The optimization problem of CR stimulation can be considered from different points of view. First of all, the stimulation should have a significant desynchronizing effect. On the other hand, the stimulation should be mild, i.e., the total amount of the delivered stimulation current should be minimized. The latter quantity depends on the stimulation strength as well as on the timing parameters of the intermittent stimulation, i.e., the lengths of the ON- and OFF-periods of the stimulation protocol. We, thus, analyze the impact of the number of stimulation sites *N*_*s*_ on these optimality criteria. The obtained results are illustrated on three different models of a neuronal network subjected to CR stimulation: the Kuramoto model of phase oscillators, a network of spiking FHN neurons, and a network of adaptive exponential integrate-and-fire bursting neurons. We show that all three models demonstrate a striking similarity in their responses to CR stimulation, which indicates the robustness of the demonstrated phenomena.

We found that the impact of the number of stimulation sites crucially depends on the spatial spread of the stimulation signal in the neuronal tissue as given by the parameter σ. If the stimulation signal is narrowly spread (small σ) we can speak about spatially selective CR stimulation where each stimulation site essentially affects neurons in its nearest proximity only. For a broad spatial signal spread (large σ) the stimulation sites perturb much larger subpopulations which may significantly overlap with each other. In our calculations the stimulation strength *I* is varied in the interval [0, *I*_max_] in order to find an optimal value *I*_opt_ leading to the strongest desynchronization characterized by minimal values of the time-averaged order parameter 〈*R*_1_〉_opt_ for continuous CR stimulation or 〈*r*〉_opt_ for intermittent CR stimulation. For the former stimulation protocol we found that an increase of the number of stimulation sites *N*_*s*_ results in a saturation of the optimal (minimal) value of the order parameter 〈*R*_1_〉_opt_. The saturation level depends on σ such that small (large) σ implies small (large) values of 〈*R*_1_〉_opt_ (Figures 3, 10). Therefore, the desynchronizing impact of the continuous CR stimulation can be improved by a moderate increase of the number of stimulation sites *N*_*s*_ for small σ. In contrast, for large σ, *N*_*s*_ has to be kept small.

For the intermittent ON–OFF CR stimulation the same conclusion can also be drawn. More precisely, for small σ a moderate increase of the number of stimulation sites *N*_*s*_ can improve all optimality criteria mentioned above (Figures 8, 11). In this case the intermittent CR stimulation admits long rest periods of the post-stimulus desynchronizing transient which are achieved for a minimal amount of the administered stimulation current. However, much larger *N*_*s*_ does not lead to any further significant improvement, and a saturation effect is observed. Moreover, for large σ the best stimulation outcome is observed for a small number of stimulation sites, e.g., *N*_*s*_ = 2, and any additional stimulation site can only worsen the desynchronizing impact of the intermittent CR stimulation.

To relate the obtained results to possible pre-clinical or clinical applications, the following values are necessary: the size *L*_exp_ of the stimulated target region where all sites are placed and the real value of the signal decay rate in the neuronal tissue σ_exp_. The latter quantity can be estimated by an analysis of the volume of tissue activated (VTA) and the corresponding voltages necessary to activate neurons at a distance *d* to the stimulation site (Chaturvedi et al., 2010). For example, the threshold activating stimulation voltage is reported to be *V*_*th*_ = *kd*^2^, where *k* ranges from 0.42 to 0.68 in the subthalamic nucleus (STN) (Chaturvedi et al., 2010). Therefore, $\sigma_{\text{exp}}=\frac{1}{\sqrt{k}}$ can be estimated as 1.2 ≤ σ_exp_ ≤ 1.5. Rescaling the real domain to the linear model segment of length *L* = 10 considered in the present study, we obtain the corresponding value of $\sigma =\sigma_{\text{exp}}\frac{L}{L_{\text{exp}}}$. If the length of the domain under stimulation is, e.g., 10mm, the value of σ lies between 1.2 and 1.5. Thus, the optimal number of stimulation sites *N*_*s*, opt_ = 2, but also for slightly larger *N*_*s*_ = 3 or 4 we expect a comparable outcome, as follows from Figure 9.

In a first approximation, we can apply our results to realistic DBS electrodes. Medtronic leads no. 3387 and no. 3389 comprise four stimulation sites, each of length 1.5 mm, and the contact spacings are 1.5 and 0.5 mm, respectively. As mentioned above, σ depends on the realistic decay rate in the neuronal tissue as well as on the size of the stimulated domain. In the framework of our approach, i.e., approximating the realistic, spatially extended stimulation contacts by points through which a monopolar stimulation is delivered (Figure 1), the Medtronic lead no. 3387 covers a target domain of length *L*_exp_ = 12 mm, whereas for the Medtronic no. 3389 lead the corresponding length *L*_exp_ = 8 mm. Assuming that the target region is large enough to embrace all stimulation sites, the values of σ range from 1.0 to 1.25 and from 1.5 to 1.9 for the former and latter electrode, respectively. Therefore, for the Medtronic no. 3387 lead from two to four sites and for the Medtronic no. 3389 lead only two remote sites enable an optimal desynchronizing effect of CR neuromodulation.

CR neuromodulation can be realized by means of a number of different stimulation modalities including electrical deep brain stimulation and sensory, e.g., acoustic stimulation for the treatment of tinnitus (Popovych and Tass, 2012; Tass and Popovych, 2012; Tass et al., 2012a,b; Adamchic et al., in press; Silchenko et al., 2013). The neuronal target regions for the former stimulation setup can be, for example, the STN or globus pallidus, whereas the non-invasive acoustic CR neuromodulation aims at a reduction of pathological synchronization in tinnitus-related auditory and non-auditory brain areas. For a more sophisticated modeling of CR deep brain stimulation one has to account for important details of the interface between the DBS electrode and the neuronal tissue and their properties as well as for the geometry and arrangement of fibers in the vicinity of the DBS electrode (Butson and McIntyre, 2006; Butson et al., 2006; Chaturvedi et al., 2010). For the acoustic CR neuromodulation, however, a precise tonotopic organization of the auditory cortex and auditory pathway has to be considered (Ehret and Romand, 1997). Furthermore, one can use models based on phase response curves (PRC) (Winfree, 1980; Ermentrout, 1996; Lücken et al., 2013) and incorporate the PRC measured either experimentally or obtained by detailed modeling of the STN, globus pallidus or cortical regions (Netoff et al., 2005; Tateno and Robinson, 2007; Tsubo et al., 2007; Stiefel et al., 2008; Schultheiss et al., 2010; Farries and Wilson, 2012a,b). Detailed neuronal models, although reflecting the richness and complexity of neuronal dynamics, are, on the other hand, so complicated and specialized that they may undermine the generality of their predictions, in particular, for other stimulation modalities and target regions. In this study we considered relatively simple models of neuronal networks and stimulation which approximate a particular realistic stimulation setup only to some extend. Nevertheless, the conclusions of the present investigation are based on fundamental properties of neuronal ensembles such as synchronization and phase resetting which are universal phenomena and can be observed under a variety of conditions, see, e.g., Winfree (1977); Best (1979), and Pikovsky et al. (2001). Ongoing oscillatory neuronal activity can be reset by both electrical and sensory stimulation (Brandt, 1997; Meissner et al., 2005; Jahangiri and Durand, 2011; Thorne et al., 2011), which serves as a basis for using different stimulation modalities for CR neuromodulation (Popovych and Tass, 2012). This supports a broad applicability of the reported results.

As mentioned in the Introduction, CR stimulation leads to sustained long-lasting after-effects on motor function in MPTP monkeys in contrast to classical HF DBS (Tass et al., 2012b). Remarkably, these sustained CR after-effects were obtained at a stimulation strength (i.e., amplitude of the single electrical pulses) equal to a third of the stimulation strength used for classical HF DBS. In contrast, delivering CR neuromodulation at a larger stimulation strength equal to that of the classical HF DBS led to only weak and considerably shorter CR after-effects. This finding is in accordance with a previous computational study where we investigated the influence of the stimulation strength and the lengths of the ON and OFF intervals (i.e., intervals with and without CR stimulation) for the intermittent CR stimulation protocol (Lysyansky et al., 2011). We found that there exists an optimal stimulation strength for CR stimulation, whereas a larger stimulation intensity can lead to a sub-optimal outcome of the stimulation. These results have now been confirmed by large-scale simulations of a precise neuronal network model of the STN and globus pallidus (Ebert, 2012) as well as experimentally in MPTP monkeys (Tass et al., 2012b). In the present study we use a similar approach and anticipate that the drawn conclusions concerning the optimal number of stimulation sites for CR stimulation may be of relevance for therapeutic effects of CR stimulation.

### Conflict of interest statement

The authors declare that the research was conducted in the absence of any commercial or financial relationships that could be construed as a potential conflict of interest.
